# Leismanicidal Activity of Propolis Collected in the Semiarid Region of Brazil

**DOI:** 10.3389/fphar.2021.702032

**Published:** 2021-07-01

**Authors:** Giani Maria Cavalcante, Celso Amorim Camara, Eva Monica Sarmento Da Silva, Mariana Silva Santos, Anderson Brandão Leite, Aline Cavalcanti Queiroz, Amanda Evelyn Da Silva, Morgana Vital Araújo, Magna Suzana Alexandre-Moreira, Tania Maria Sarmento Silva

**Affiliations:** ^1^ Phytochemical Bioprospecting Laboratory, Chemistry Department, Rural Federal University of Pernambuco, Pernambuco, Brazil; ^2^ Zootechnical Collegiate, Federal University of the São Francisco Valley, Pernambuco, Brazil; ^3^ Pharmacology and Immunity Laboratory, Institute of Biological and Health Sciences, Federal University of Alagoas, Alagoas, Brazil; ^4^ Microbiology, Immunology and Parasitology Laboratory, Campus Arapiraca, Federal University of Alagoas, Alagoas, Brazil

**Keywords:** flavonoids, propolis, leishmanicidal activity, *Leishmania (Leishmania) amazonensis*, *Leishmania (Leishmania) chagasi*

## Abstract

**Objective:** The aim of the current study is to investigate the chemical composition, cytotoxic effect, and leishmanicidal activity of propolis collected in the semi-arid region of Bahia, Brazil.

**Methods:** EtOH extract, hexane, EtOAc and MeOH fractions from propolis were analyzed by ultra-performance liquid chromatography coupled with diode array detector and quadrupole time-of-flight mass spectrometry. The identification was based on the exact mass, general fragmentation behaviors and UV absorption of the flavonoids. The *in vitro* cytotoxic effect and leishmanicidal activity of ethanolic extract, hexane, ethyl acetate, and methanolic fractions of propolis were evaluated.

**Results:** Five triterpenes and twenty-four flavonoids were identified. The propolis did not present toxicity to the host cell up to the maximum concentration tested. In addition, all tested samples showed statistically significant activity against promastigotes of *Leishmania chagasi* and *Leishmania amazonensis.* Regarding the activity against amastigote forms of *L. amazonensis*, the hexane fraction, presented statistically significant activity with IC_50_ of 1.3 ± 0.1 μg/ml.

**Conclusion:** The results support the idea that propolis can be used for future antileishmania studies.

## Introduction

Leishmaniasis is one of the six major parasitic diseases targeted by the World Health Organization. It is endemic in 98 countries and more than 350 million people are at risk of infection. The disease causes 20,000–40,000 deaths per year globally (Sasidharan and Saudagar, 2021).

In humans, the clinical forms of leishmaniasis are broadly categorized into cutaneous leishmaniasis (CL) and visceral leishmaniasis (VL), and clinical manifestations depend on the pathogenicity of the parasite, which differs among species, and the genetically determined cell-mediated immune response of the human host (Singh et al., 2012).

Current leishmaniasis treatment strategy is based on chemotherapy with some attempts at immunotherapy, but the combination of factors including the development of parasite resistance to drug therapies, the absence of vaccines, problems with vector control, among other factors, has rendered the treatment options for leishmaniasis limited (Taslimi et al., 2018).

Pentavalent antimonials are the standard first line treatment, but the emergence of resistance has limited their usefulness. Alternative chemotherapeutic treatments with amphotericin B and its lipid formulation, miltefosine, and paromomycin are available but their use is limited either due to toxicity or the high cost of treatment (Braga, 2019).

Consequently, it is of the utmost importance to discover effective drugs and new drug targets for the treatment of leishmaniasis. Propolis has long been the subject of pharmaceutical interest because of its potent biological functions, such as antimicrobial, anti-inflammatory, anti-protozoan, and antitumoral activities (Popova et al., 2005; Dantas et al., 2006; Jin et al., 2008; Szliszka et al., 2013). The propolis resin is complex, and its common constituents include polyphenols (flavonoids, phenolic acids, and their esters, terpenoids, and steroids (Tran et al., 2012). Its chemical composition depends on the plant or plants from which the resin is collected, the geographical location, and the associated flora (Popova et al., 2005; Ishida et al., 2011).

Therefore, there has been an interest in the composition and biological properties of this natural product. Extensive investigations have been conducted with propolis from different countries in temperate and tropical areas (Sforcin, 2016); however, a limited number of investigations have been conducted to study the palynological analysis (Matos et al., 2014), chemical composition (Valcic et al., 1998; Hernandez et al., 2007) and biological activities of propolis from semiarid regions (Russo et al., 2004).

In our continuing studies of the chemical and biological activity of *Apis* and *Melipona* bee products (Silva et al., 2006; Silva et al., 2009; Freire et al., 2012; Silva et al., 2013; Souza et al., 2013; Souza et al., 2014; Sousa-Junior et al., 2019; Sousa-Fontoura et al., 2020), we investigated the chemical composition of propolis by ultra-performance liquid chromatography coupled with diode array detector and quadrupole time-of-flight mass spectrometry (UPLC-DAD-QTOF-MS/MS), and we also analyzed the cytotoxic effect and leishmanicidal activity of the ethanolic extract, hexane, ethyl acetate, methanolic fractions of propolis from semi-arid region of Bahia, Brazil.

## Methods

### Propolis Sample and Extraction

Propolis was collected in Brazil, State of Bahia, municipality of Casa Nova, which is an area of the Caatinga, a Brazilian biome. The propolis (79.4 g) was successively extracted with ethanol (EtOH) in an ultrasonic bath and evaporation was performed using a rotary evaporator in a vacuum at 40°C. 20 g dry extract was dissolved in methanol:water (1:1, MeOH) and successively fractionated with hexane (hexane, 7.4 g) and ethyl acetate (EtOAc, 9.1 g). The amount of MeOH fraction was 1.1 g. The EtOH extract, hexane, EtOAc, and MeOH fractions, were subjected to UPLC-PDA-QTOF-MS analysis.

### Analysis of Propolis by Ultra-Performance Liquid Chromatography Coupled With Diode Array Detector and Quadrupole Time-of-Flight Mass Spectrometry

The analysis was performed using a XEVO-G2XSQTOF mass spectrometer (Waters, Manchester, United Kingdom) connected to an ACQUITY UPLC system (Waters, Milford, MA, United States ) via an electrospray ionization (ESI) interface. Chromatographic separation of the compounds was performed on the ACQUITY UPLC with a conditioned autosampler at 4 C, using an Acquity BEH C18 column (150 mm × 2.1 mm i.d., 1.7 μm particle size). The column temperature was maintained at 40°C. The mobile phase consisted of water with 0.1% formic acid (solvent A) and acetonitrile (solvent B), and it was pumped at a flow rate of 0.4 ml min^−1^. The gradient elution program was as follows: 0–5 min, 5–10% B; 5–9 min, 10–95% B. The injection volume was 10 μl. MS analysis was performed on a Xevo G2 QTOF (Waters MS Technologies, Manchester, United Kingdom), a quadrupole time-of-flight tandem mass spectrometer coupled with an electrospray ionization source in the negative ion mode. The scan range was from 50 to 1,200 *m/z* for data acquisition. In addition, MS^E^ experiments were carried out which allow precursor and product ion data to be acquired in one injection. The source conditions were as follows: capillary voltage, 2.0 kV; sample cone, source temperature, 100 C; desolvation temperature 250°C; cone gas flow rate 20 Lh^−1^; desolvation gas (N_2_) flow rate 600 Lh^−1^. All analysis were performed using the lockspray, which ensured accuracy and reproducibility. Leucine-encephalin (5°ngml^−1^) was used as a standard or reference compound to calibrate the mass spectrometers during analysis. All the acquisition and analysis of data were controlled using Waters MassLynx v 4.1 software.

### Cells and Parasites

J774.A1 macrophages were cultured *in vitro* in RPMI-1640 medium supplemented with 10% FBS, 2 mM *l*-glutamine, nonessential amino acids and pyruvate at 37°C with 95% humidity and 5% CO_2_ in an incubator. Two strains of *Leishmania* were used in the present study: *Leishmania (Leishmania) amazonensis* [MHOM/BR/77/LTB0016] and *Leishmania* (*Leishmania) infantum chagasi* [MCAN/BR/89/BA262]. They were maintained *in vitro* at 26°C in Schneider’s medium supplemented with 10% FBS, gentamycin (1 mg/L), *l*-glutamine (2 mM), and 2% sterile human urine.

### Cytotoxicity Assay

J774.A1 macrophages were seeded (1 × 10^5^ cell/well) in 96-well plates with 100 ml of RPMI-1640 medium and incubated at 37°C for 1 h. After this period, the cells were treated with 0.1, 1, 10, and 100 μg/ml of EtOH extract and organic fractions hexane, EtOAc, MeOH; 0.1, 1, 10, and 100 µM of pentamidine (all treatments performed in triplicate) previously diluted in RPMI-1640 medium with 0.1% dimethyl sulfoxide (DMSO). The plates were maintained in a 5% CO_2_ incubator at 37°C for 48 h. Cells were also cultured in media free of compounds, or media with 0.1% DMSO (vehicle control). Thereafter, the supernatant was removed and cells were incubated with 3-(4,5-dimethylthiazol-2-yl)-2,5-diphenyltetrazolium bromide (MTT) (0.5 mg/ml) for 1 h in the dark at 37°C. The MTT solution was removed, cells were resuspended in 100 ml of 0.1% DMSO, and the absorbance was measured using an ELISA reader at 550 nm (Hussain et al., 1993).

### Leishmanicidal Assay

Cells (1 × 10^5^/well) of promastigotes forms of *L. amazonensis* and *L. chagasi* were cultured in Schneider’s medium supplemented with 10% FBS and 2% human urine, in the presence of various concentrations of EtOH extract, hexane, EtOAc and MeOH fractions (0.1, 1, 10 and 100 μg/ml), and pentamidine (0.1, 1, 10, and 100 µM) in triplicate for 48 h at 26°C. Cells were also cultured in a medium free of substances, a vehicle (basal growth control) or with 0.1% DMSO (vehicle control). After 48 h, the viability of the promastigotes forms was analyzed using the MTT assay. MTT (20 μL) was added to each well and incubated at 37°C for 2 h with 95% humidity and 5% CO_2._ Formazan extraction was performed using 120 μL of isopropanol and left at room temperature for 2 h. The absorbance was measured using an ELISA reader at 550 nm (Moraes et al., 2014). The intracellular amastigote assay was performed in 24-well microplates with rounded coverslips on the bottom. J774.A1 macrophages were seeded at a density of 3 × 10^5^ cells/well and maintained for 1 h in 5% CO2 at 37°C for adhesion in a humidified atmosphere of 95% air and 5% CO_2_ at 37°C. Afterward, the cells were infected *in vitro* with promastigote forms of *L. amazonensis* and *L. chagasi* at a ratio of 1:10 for 6 h in a humidified atmosphere of 95% air and 5% CO_2_ at 37°C. The parasites in the supernatant were removed by washing, and various concentrations of EtOH extract, hexane, EtOAc, and MeOH fractions (0.1, 1, 10, and 100 μg/ml) and pentamidine (0.1, 1, 10, and 100 µM) were added and maintained at 37 C in 5% CO_2_ for 48 h. The glass coverslips were fixed with methanol, stained with May-Grünwald-Giemsa, and intracellular amastigotes were counted (one hundred macrophages were evaluated per glass coverslip). Data were expressed as infection index (percentage of infected macrophages multiplied by the average number of amastigotes per macrophage) (Hussain et al., 1993).

### Statistical Analysis

Results were expressed as the mean ± SEM of an experiment in triplicate. The tests were performed with controls: media free from compounds, a vehicle (basal growth control), or media with 0.1% DMSO (vehicle control). Significant differences between the treated and control groups were evaluated using ANOVA and Dunnett *post-hoc* tests by Graph Pad Prism 5.0 software, and 95% confidence intervals were included.

## Results

**FIGURE 1:**
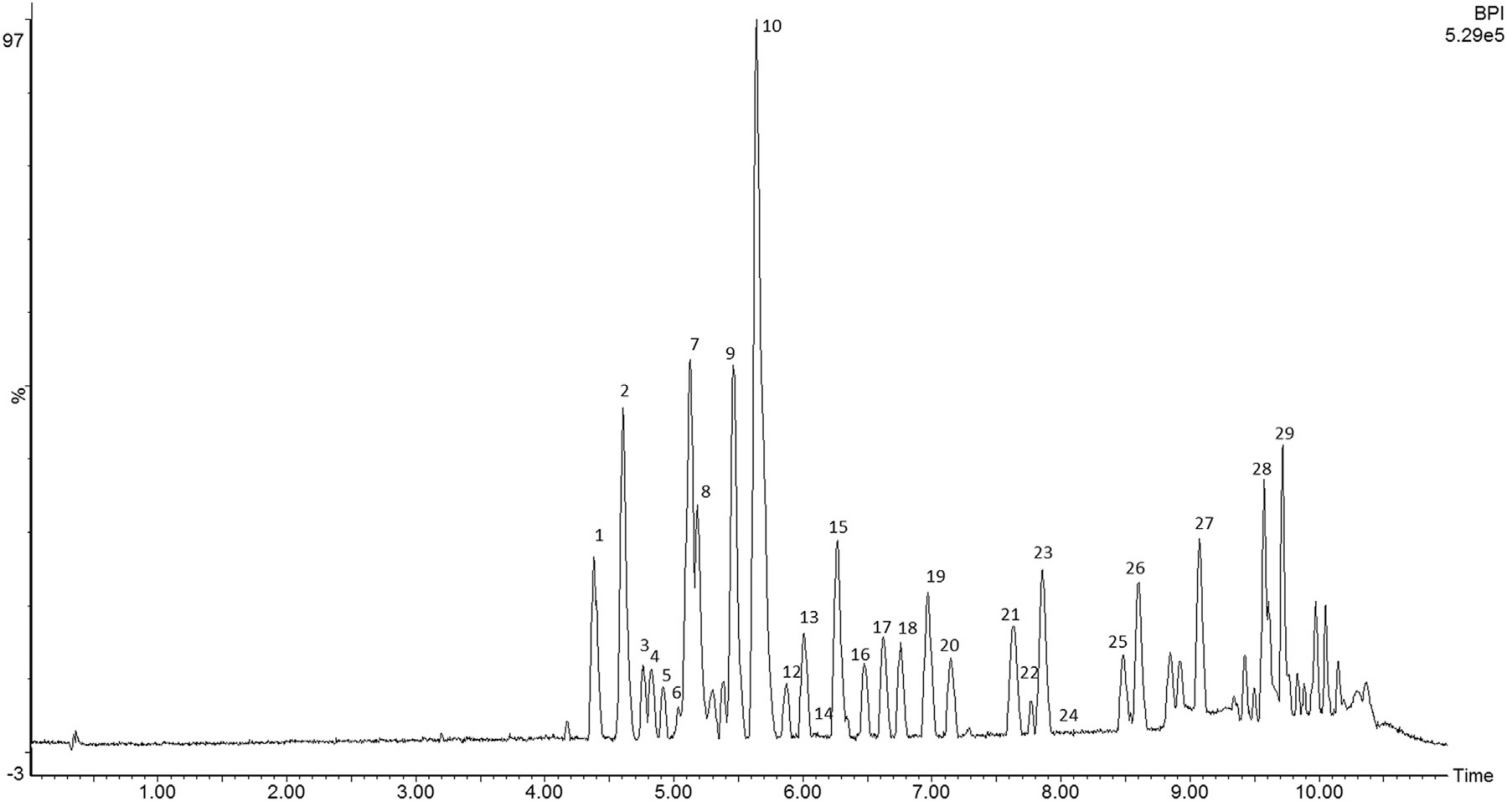
Base peak ion chromatogram of propolis EtOH extract obtained by an MS^E^ data collection technique method (UPLC-qTOF/MS^E^) in negative mode.

**FIGURE 2:**
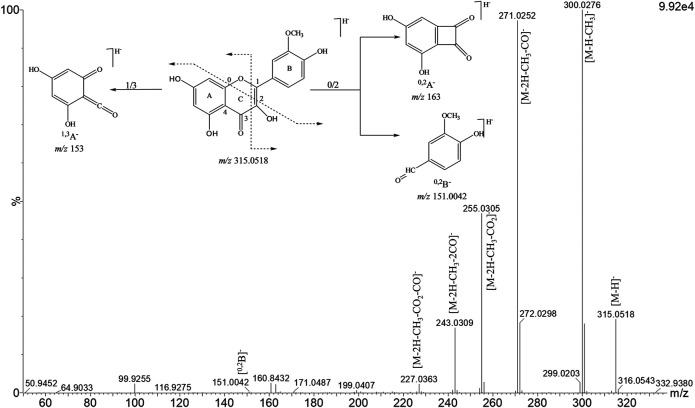
Product ion spectrum of flavonoid 8 (Isorhamnetin).

The compounds were tentatively identified by ultra-performance liquid chromatography coupled with diode array detector and quadrupole time-of-flight mass spectrometry (UPLC-DAD-QTOF-MS/MS), as flavonoids (flavonol/flavone, flavanone, and chalcones) based on their characteristic UV-Vis (flavonoids) spectra peaks and mass detection as well as the accurate mass measurement of the precursor and product ions. Terpenes were suggested by the absence of absorption in the UV spectra, in addition to the high-resolution mass spectra. Figure 1 shows the base peak ion (BPI) chromatogram of the ethanol extract from propolis. The groups of flavonoids could be distinguished based on their UV-vis spectra which resembled that of flavone/flavonol (with two absorbance maxima near 250–260 and 350), flavanone (maximum near 280–290 nm), and chalcones (maxima near 242 and 364 nm). All the detected compounds were listed in Table 1. The analysis allowed the identification of 24 flavonoids including 13 flavonols/flavones (1, 2, 3, 5, 6, 7, 8, 9, 10, 11, 15, 16, 17, 18, 20, 21, 22 and 23), 2 chalcones (12 and 24) and 4 flavanones (4, 13, 14 and 19). The flavonoids 1, 2, 4, 8, and 19 were compared with standard samples. It is interesting to note that the identified flavonoids present only six main nuclei, and the difference was the presence of the methoxyl groups in the flavonoids. The fragments found for flavonoids are similar to the flavonoids reported by Santisteban et al., 2019. Methoxylated flavonoids lost preferentially nCH_3_^•^, yielding the characteristic fragments of [M - H-n × 15]^+^. These methyl groups have been cleaved as methyl radicals in position-independent manners. Other fragments, such as the neutral loss of CH_4_ (14 Da), CO_2_ (44 Da), and CO (28 Da), were frequently detected. If there are protons adjacent the methoxy on the flavonol rings, intra-molecule proton transfer can occur, and one molecule of methane can be eliminated (Ma et al., 2013). Retro-Diels-Alder (RDA) reactions played key roles in the identification of flavonoids and their derivatives (Demarque et al., 2016). Consider isorhamnetin 8) as an example, the characteristic ion of methoxylated was observed by prominent ion at *m/z* 300.0292 [M-H-CH_3_]^−^ by the loss of 15 Da (CH_3_) from the protonated molecular ion at *m/z* 315.0518 [M-H]^−^ (Figure 2). The abundant ion at *m/z* 271.0265 [M-2H-CH_3_-CO]^−^, might be obtained by the loss of CO (28 Da) from *m/z* 300.0292. Subsequent neutral loss of CO_2_ or CO and gave a secondary abundant ion at *m/z* 255.0305 [M-2H-CH_3_-CO_2_]^−^ and *m/z* 243.0309 [M-2H-CH_3_-2CO]^−^, respectively and another ion at *m/z* 227.0663 [M-2H-CH_3_-CO-CO_2_]^−^. Additionally, the fragmentation pathways of RDA cleavage of the C-ring generated relative low abundance ion at *m/z* 151.0042 [^0,2^B]^−^ (Figure 2). The compounds shown at the end of the chromatogram of EtOH extract (Figure 1) can be identified as triterpenes because they do not absorb in the UV region and had a molecular constitution typical of triterpenes derivatives (25–29). The main product ions observed in the mass spectra were due to the loss of CH_3_ (15 Da) and H_2_O (18 Da). These compounds are isomers and were not completely identified.

**TABLE 1:** Characterization of compounds from propolis EtOH extract, hexane, EtOAc and MeOH fractions analyzed by UPLC-DAD-ESI-qTOF-MS^E^ in negative mode.

	RT (min)	λ_max_ (nm)	[M-H]^-^ (*m/z*)	[M-H]^-^ (*m/z*) Calculated	MS^2^ Fragments identification	Tentative identification^a^
1^e^	4.38	359	301.0350	301.0353	271.0274 [M-2H-CO]^−^, 193.0133 [M-H-B]^-^, 151.0045 [^1,3^A]^−^	Quercetin^a,c,d^
2^e^	4.60	356	315.0512	315.0510	300.0208 [M-H-CH_3_]^−^, 271.0251 [M-2H-CH_3_-CO]^−^, 255.0307 [M-2H-CH_3_-CO-CH_4_]^−^, 151.0044, 151.0045 [^1,3^A]^−^	3-*O*-methyl-quercetin^a,c,d^
3	4.76	345	345.0615	345.0615	330.0388 [M-H-CH_3_]^−^, 315.0159 [M-H-2CH_3_]^−^, 287.0208 [M-H-2CH_3_-CO]^−^	Myricetin dimethyl-ether^a,c^
4^e^	4.82	287	271.0610	271.0611	151.0029 [^1,3^A]^−^, 119.0505 [^0,2^B]^−^	Naringenin^a,c^
5	4.91	339	269.0455	269.0455	151.0037 [^1,3^A]^−^	Trihydroxy-flavone^a,c^
6	5.02	364	285.0405	285.0404	257.0524 [M-H-CO]^−^	Tetrahydroxy-flavone^a,c^
7	5.48	346	299.0563	299.0561	284.0323 [M-H-CH_3_]^−^, 227,0359 [M-2H-CH_3_-2CO]^−^, 151.0058 [^1,3^A]^−^	Trihydroxy-methoxy-flavone^a,c,d^
8^e^	5.16	346	315.0518	315.0510	300.0276 [M-H-CH_3_]^−^, 271.0265 [M-2H-CH_3_-CO]^−^, 255.0305 [M-2H-CH_3_-CO_2_]^−^, 243.0309 [M-2H-CH_3_-2CO]^−^, 227.0663 [M-2H-CH_3_-CO-CO_2_]^−^, 151.0042 [^0,2^B]^−^	Isorhamnetin^a,c^
9	5.44	346	299.0563	299.0561	284.0302 [M-H-CH_3_]^−^, 255.0294 [M-2H-CH_3_-CO]^−^, 227.0347 [M-2H-CH_3_-2CO]^−^	Trihydroxy-methoxy-flavone^a,c,d^
10	5.64	345	329.0662	329.0667	314.0409 [M-H-CH_3_]^−^, 299.0195 [M-H-2CH_3_]^−^, 271.0248 [M-H-2CH_3_-CO]^−^, 243.0309 [M-H-2CH_3_-2CO]^−^	Quercetin dimethyl ether^a,c,d^
11	5.65	345	329.0660	329.0667	314.0409 [M-H-CH_3_]^−^, 299.0195 [M-H-2CH_3_]^−^, 271.0248 [M-H-2CH_3_-CO]^−^, 243.0309 [M-H-2CH_3_-2CO]^−^	Quercetin dimethyl ether^a,c^
12	5.83	369	285.0768	285.0768	270.0570 [M-H-CH_3_]^−^	Hydroxy-methoxy-chalcone
13	5.98	286	301.0718	301.0717	285.0396 [M-2H-CH_3_]^−^, 165.0206 [^0,2^A]^−^, 135.0457 [^0,2^A-CO]^−^	Trihydroxy-methoxy-flavanone^a,c^
14	6.07	275	269.0826	269.0819	241.7910 [M-H-CO]^−^	Hydroxy-methoxy-flavanone^a,c^
15	6.23	333	313.0716	313.0716	298.0477 [M-H-CH_3_]^−^, 283.0245 [M-H-2CH_3_]^−^, 255.0307 [M-H-2CH_3_-CO]^−^	Dihydroxy-dimethoxy-flavone^a,c^
16	6.44	356	315.0513	315.0510	300.0227 [M-H-CH_3_]^−^, 135.9030 [^0,2^A-CO]^−^	Tetrahydroxy-methoxy-flavone^a,c^
17	6.59	360	329.0666	329.0667	314.0409 [M-H-CH_3_]^−^, 299.0195 [M-H-2CH_3_]^−^, 271.0248 [M-H-2CH_3_-CO]^−^, 243.0309 [M-H-2CH_3_-2CO]^−^	Trihydroxy-dimethoxy-flavone^a,c^
18	6.72	339	343.0822	343.0823	328.0589 [M-H-CH_3_]^−^, 313.0352 [M-H-2CH_3_]^−^, 298.0122 [M-2H-3CH_3_]^−^, 285.0414 [M-2H-3CH_3_-CO]^−^	Dihydroxy-trimethoxy-flavone^a,c^
19^e^	6.93	287	285.0765	285.0768	269.0383 [M-2H-CH_3_]^−^, 119.0505 [^0,2^B]^−^	7-*O*-methyl naringenin^a,b,c^(sakuranetin)^e^
20	7.11	339	283.0618	283.0612	268.0373 [M-H-CH_3_]^−^, 240.0427 [M-H-CH_3_-CO]^−^	Dihydroxy methoxy flavone^a,b,c^
21	7.32	346	313.0721	313.0718	298.0477 [M-H-CH_3_]^−^, 283.0245 [M-H-2CH_3_]^−^, 255.0307 [M-H-2CH_3_-CO]^−^	Dihydroxy-dimethoxy-flavone^a^ ^,^ ^c^
22	7.82	345	313.0718	313.0718	298.0477 [M-H-CH_3_]^−^, 283.0245 [M-H-2CH_3_]^−^, 255.0307 [M-H-2CH_3_-CO]^−^	Dihydroxy-dimethoxy-flavone^a,b,c^
23	7.84	339	343.0829	343.0823	328.0586 [M-H-CH_3_]^−^, 313.0354, [M-H-2CH_3_]^−^, 285.0407 [M-H-2CH_3_-CO]^−^, 270.0168 [M-H-3CH_3_-CO]^−^	Dihydroxy-trimethoxy-flavone^a,b,c^
24	7.93	363	269.0822	269.0819	235.9273 [M-2H-CH_3_-H_2_O]^−^	Dihydroxy-methoxy-chalcone^a,c^
25	8.48		515.3223	515.3226	469.3183 [M-H-CO-H_2_O]^−^	Triterpene^a,b^
26	8.60		515.3223	515.3226	485.3106 [M-H-2CH_3_]^−^, 467.3019 [M-H-2CH_3_-H_2_O]^−^, 451.3044 [M-H-2CH_3_-H_2_O-CH_4_]^−^	Triterpene^a,b^
27	8.92		499.3273	499.3276	453.3282 [M-H-CO-H_2_O]^−^	Triterpene^a,b^
27	9.57		515.3223	515.3226	497.3112 [M-H- H_2_O]^−^, 467.3008 [M-H-2CH_3_]^-^	Triterpene^a,b^
29	9.72		499.3273	499.3276	469.3171 3,008 [M-H-2CH_3_]^-^	Triterpene^a,b^

a Found in the EtOH extract.

b Found in the hexane fraction.

c Found in the EtOAc fraction.

d Found in the MeOH fraction.

e Compared to a previously isolated and identified standard.

The *in vitro* leishmanicidal activity was evaluated with extract and fractions of propolis. The results presented in Table 2 show the effects of the ethanol extract (EtOH), hexane fraction (hexane), ethyl acetate fraction (EtOAc), methanolic fraction (MeOH), and pentamidine against J774.A1 macrophages using the MTT assay. After 48 h of incubation, pentamidine showed deleterious activity to the host cell with maximum cytotoxicity of 93.8 ± 0.7% and IC_50_ of 44.1 ± 0.8 µM. However, the extract, and fractions not showed deleterious activity to the host cell, presenting as promising substances for the other assays. Orsi et al. (2005), observed that Brazilian propolis was not toxic to macrophages, and using cytotoxicity assays, Blonska et al. (2004),observed that the ethanolic extract of propolis was not toxic to these macrophage cells.

**TABLE 2:** Determination of the cytotoxicity of pentamidine, EtOH extract, and hexane, EtOAc, MeOH fractions, of propolis from the semi-arid region of Bahia, Brazil against J774.A1 cells using the MTT assay.

Treatment	IC50 (µM)^a^	Maximum Cytotoxicity (%)^b^
Pentamidine	44.1 ± 0.8 µM	93.8 ± 0.7**+
EtOH	>100 μg/ml	ND
Hexane	>100 μg/ml	ND
EtOAc	>100 μg/ml	ND
MeOH	>100 μg/ml	ND

a Inhibitory concentration 50 (IC_50_) calculated by concentration-response curves toxic.

b Mean ± standard error of the mean maximum cytotoxicity in triplicates of a representative experiment. The values of maximum effect were considered significant when **p < 0.01 compared to the 0.1% DMSO group. IC_50_ is the concentration required to give 50% inhibition. ND: Not determined. Maximum cytotoxicity compared to the DMSO group.

The leishmanicidal activity of EtOH extract; hexane, EtOAc, MeOH fractions, and pentamidine against the promastigote forms of *L. amazonensis* and *L. chagasi* was assessed *in vitro*. The inhibitory effects and IC_50_ values are shown in Table 3. Hexane, EtOAc, and MeOH fractions exhibited the most significant leishmanicidal activity to *L. amazonensis*. The EtOH extract, hexane and EtOAc fractions exhibited the most significant leishmanicidal activity to *L. chagasi.* Thus, it is observed that EtOH extract and EtOAc fraction showed the best inhibitory activity against the growth of promastigote forms of *L. amazonensis* and *L. chagasi*, respectively. Moreover, EtOAc and MeOH fractions showed the anti-amastigote activity against *L. amazonensis* and *L. chagasi*, respectively.

**TABLE 3:** Effects of pentamidine, EtOH extract; hexane, EtOAc, MeOH fractions of propolis from the semi-arid region of Bahia, Brazil against promastigote forms of *L. amazonensis* and *L. chagasi.*

Treatment	*L. amazonensis*		*L. chagasi*	
	IC_50_ ^a^	Maximum effect (%)^b^	IC_50_ ^a^	Maximum effect (%)^b^
Pentamidine	4.7 ± 1.0 µM	97.8 ± 0.7***	6.1 ± 0.3 µM	96.5 ± 0.4***
EtOH	7.1 ± 1.1 μg/ml	94.3 ± 1.9***	4.2 ± 0.9 μg/ml	71.5 ± 2.3**
Hexane	8.2 ± 2.6 μg/ml	96.1 ± 0.2***	6.2 ± 1.9 μg/ml	68.6 ± 7.5**
EtOAc	5.8 ± 0.5 μg/ml	96.6 ± 1.2***	6.6 ± 0.3 μg/ml	71.5 ± 2.4**
MeOH	5.6 ± 1.0 μg/ml	95.8 ± 1.3***	7.3 ± 0.9 μg/ml	53.9 ± 7.5**

a Inhibitory concentration 50 (IC_50_) calculated by concentration-response curves toxic.

b Mean ± standard error of the mean maximum cytotoxicity in triplicates of a representative experiment. Differences with **p < 0.01 and ***p < 0.001 were considered significant in relation to the 0.1% DMSO group. IC_50_ is the concentration required to give 50% inhibition. The maximum effect was at concentrations 10 µg/ml.

## Discussion

The presence of flavonoids and terpenes were identified in the active EtOH extract, hexane and EtOAc fractions. In this study, the chemical profile of the flavonoids is similar to that found for the geopropolis samples of stingless bees collected in the semi-arid region (Sousa-Junior et al., 2019; Sousa-Fontoura et al., 2020). However, additional studies need to be carried out to know the origin of the plant species from which bees collect the resin in the semi-arid region of Brazil.

Propolis is an important source of substances applied in medicine due to its pharmacological activities (Potin et al., 2008) and wide range of biological properties, including leishmanicidal activity (Duran et al., 2008; Potin et al., 2008; Nina et al., 2016). In another study, the ethanolic extract of propolis showed leishmanicidal activity against *L. tropica* at concentrations of 250, 500 and 750 μg/ml, and statistically significant differences in cell counts were observed compared to the control group (*p* < 0.05) (Duran et al., 2008), whereas the fractionated propolis-rich phenolic showed maximum efficacy against *L. amazonenis* (IC_50_ = 12.1 μg/ml) and *L. brasiliensis* (IC_50_ = 10.9 μg/ml) (Nina et al., 2016). Extracts of propolis were tested against protozoal pathogens, including *Crithidia fasciculata* a close relative of *Crithidia mellificae*, a parasite of bees. High levels of activity were obtained for all the samples of different extracts. In the case of *C. fasciculata* highest activity was associated with flavonoids methyl ethers of galangin and pinobanksin (Alotaibi et al., 2019). According to Ruiz-Gonzalez and Brown (2006), the spread of the protozoal infection occurs via feces, coating the surfaces in the hive with propolis that is active against trypanosomiasis could prevent transmission.

The results of the evaluation of leishmanicidal activity against intracellular forms of *L. amazonensis* revealed that hexane fraction induced the growth inhibition of amastigotes forms by 60.4 ± 1.4%, 63.4 ± 1.7%, 59.4 ± 1.9%, and they had IC_50_ values of 1.3 ± 0.1 μg/ml, 1.9 ± 1.5 µM, and1.4 ± 0.9 µM, respectively (Table 4).

**TABLE 4:** Effects of pentamidine, EtOH extract; hexane, EtOAc, MeOH fractions of propolis from the semi-arid region of Bahia, Brazil, against amastigote forms of *L. amazonensis* and *L. chagasi*.

Treatment	*L. amazonensis*		*L. chagasi*	
	IC_50_^a^	Maximum effect (%)^b^	IC_50_^a^	Maximum effect (%)^b^
Pentamidine	1.5 ± 1.2 µM	69.2 ± 0.4**	1.7 ± 0.5 µM	75.3 ± 2.0**
EtOH	>100 μg/ml	ND	>100 μg/ml	ND
Hexane	1.3 ± 0.1 μg/ml	60.4 ± 1.4**	>100 μg/ml	ND
EtOAc	>100 μg/ml	ND	>100	ND
MeOH	>100 μg/ml	ND	7.2 ± 0.4 μg/ml	55.2 ± 1.0*

Data are reported as the mean ± standard error of the mean, S.E.M. The values of efficacy were considered significant when *p < 0.05, **p < 0.01, and***p < 0.01 compared to the 0.1% DMSO group. IC_50_ is the concentration required to give 50% inhibition; ND: not determined; Maximum cytotoxicity compared to the DMSO group. The maximum effect was at concentrations 10 μg/ml to fractions.

According to Ayres et al. (2007), the ethanolic extract of propolis presented a direct effect on the amastigote forms of *L. amazonensis*, up to 96 h, at a concentration of 25 μg/ml.

Despite being widely studied, propolis remains an important source of metabolites for the treatment of diseases. Moreover, leishmaniasis is a current public health issue with a high global social impact. The currently available treatments for this disease are limited in terms of toxicity, and they have variable efficacy and high costs, which potentiates the results obtained in this study.

## Conclusion

To the best of our knowledge, this is the first study which describes the chemical composition and biological activity of propolis collected from the semiarid region in Northeast Brazil. The extract, and fractions from propolis anti-leishmanial effects. The observed effects could be associated with the presence of flavonoids. The chemical and biological characterization of the semiarid region could be important for the development of alternative treatment strategies against *Leishmania* sp.

## Data Availability

The raw data supporting the conclusions of this article will be made available by the authors, without undue reservation.
